# Caspase-Like Activities Accompany Programmed Cell Death Events in Developing Barley Grains

**DOI:** 10.1371/journal.pone.0109426

**Published:** 2014-10-06

**Authors:** Van Tran, Diana Weier, Ruslana Radchuk, Johannes Thiel, Volodymyr Radchuk

**Affiliations:** Institute of Plant Genetics and Crop Plant Research (IPK), Gatersleben, Germany; IISER-TVM, India

## Abstract

Programmed cell death is essential part of development and cell homeostasis of any multicellular organism. We have analyzed programmed cell death in developing barley caryopsis at histological, biochemical and molecular level. Caspase-1, -3, -4, -6 and -8-like activities increased with aging of pericarp coinciding with abundance of TUNEL positive nuclei and expression of *HvVPE4* and *HvPhS2* genes in the tissue. TUNEL-positive nuclei were also detected in nucellus and nucellar projection as well as in embryo surrounding region during early caryopsis development. Quantitative RT-PCR analysis of micro-dissected grain tissues revealed the expression of *HvVPE2a*, *HvVPE2b*, *HvVPE2d*, *HvPhS2* and *HvPhS3* genes exclusively in the nucellus/nucellar projection. The first increase in cascade of caspase-1, -3, -4, -6 and -8-like activities in the endosperm fraction may be related to programmed cell death in the nucellus and nucellar projection. The second increase of all above caspase-like activities including of caspase-9-like was detected in the maturating endosperm and coincided with expression of *HvVPE1* and *HvPhS1* genes as well as with degeneration of nuclei in starchy endosperm and transfer cells. The distribution of the TUNEL-positive nuclei, tissues-specific expression of genes encoding proteases with potential caspase activities and cascades of caspase-like activities suggest that each seed tissue follows individual pattern of development and disintegration, which however harmonizes with growth of the other tissues in order to achieve proper caryopsis development.

## Introduction

Programmed cell death (PCD) is a highly regulated cellular suicide process essential for growth, development and survival of all eukaryotic organisms. In plants, developmental PCD accompanies the entire life cycle: seed germination [1], aerenchyma formation [2], tracheary and sieve element differentiation [3], [4], leaf shape formation [5], reproduction [6], [55], somatic embryogenesis [54], [56], senescence [7] and responses against abiotic stresses and pathogens [8].

Development of cereal seeds, including barley grains, is largely accompanied by regular cell death. Mature cereal grains, a main source for human food, domestic animal feed and many industrial applications, consist mainly of dead material. Only the relatively small embryo and aleurone layer are still alive in ripe grains. The regular cell degeneration in cereal caryopses starts soon after fertilization with disintegration of antipodal and synergid cells. Embryo and endosperm develop within the maternal tissues nucellus, inner and outer integuments, and pericarp, which represent the bulk of the early grain. The pericarp can be divided in exocarp or epidermis, mesocarp (representing the majority of pericarp cells) and endocarp or chlorenchyma [9]. The nucellus degenerates within several days after flowering (DAF) providing space and nutrients for the early endosperm [10]–[12]. Only the nucellar region opposite to the main vascular bundle stays alive and differentiates into the nucellar projection, which functions as a transfer tissue to deliver the assimilates to the endosperm [13]. The assimilate release from the nucellar projection requires PCD of the tissue [11], [14]. The growth of the endosperm takes place at the expense of pericarp which largely degenerates till 12 DAF [12] with the exception of the region surrounding the main vascular bundle. Also cells of the starchy endosperm undergo PCD during later development [15], [16].

Little is known about molecular mechanisms underlying PCD in plants. In animals, classical PCD is executed by specific proteases, called caspases, with characteristic cysteines in the catalytic domain. Caspases cleave target peptides at C-terminal after aspartate [17], [18] and are involved in apoptosis and development [17]. PCD execution in plants is also often associated with caspase-like activities [19]. Caspase-1, caspase-3 and caspase-6-like activities were detected in the degenerating nucellus of *Sechium edule* [20]. In the developing barley grains, several caspase-like activities were measured at 10 and 30 days after flowering [21]. Taking into account that diverse and often contradictory processes happens simultaneously (i.g., degeneration of pericarp coincides with endosperm expansion) in the caryopses, measurements of caspase activities in distinct tissues over whole development are necessary to detect PCD processes in the developing grain. While caspase activities have been detected in plants, sequences similar to animal caspases are not present in plant genomes. The metacaspases with weak structural similarity to caspases are likely involved in PCD [22], but do not execute caspase-specific proteolytic activity recognizing substrates with either lysine or arginine instead of aspartate [23], [24]. Other plant proteases with limited similarity to animal caspases display caspase-like activities and are involved in diverse types of PCD. In particular, vacuolar processing enzyme (VPE), also called legumain, is responsible for caspase-1 activity in plants [25]–[27]. The 20S proteasome, composed of many α and β subunits, executes caspase-3 activity during xylem development [3] and in response to biotic stress [28]. It has been also shown that the β1 subunit (PBA) and, possibly, the β2 subunit (PBB) provide caspase-3 activity whereas the β5 subunit of the 20S proteasome does not [3]. The subtilisin-like protease called phytaspase has been found to exhibit caspase-6 activity in tobacco and rice [29]. The saspase from *Avena sativa*, which is very similar to phytaspase, is also uses caspase-6 substrates [30]. The caspase-2 and caspase-4 like activities have not been reported in plants so far [31]. With exclusion of VPE genes [12], [27], other genes encoding proteases with the respective caspase-like activity have not been described so far in barley.

While the PCD events have been well documented in maternal seed parts of early developing barley grain [12], there is no information about timing and localization of PCD during later seed development. Here we have revealed temporal and spatial PCD patterns over whole barley grain development using the TUNEL assay. Caspase-like activities in separated pericarp and endosperm fractions have been investigated and expression of candidate genes potentially responsible for these activities was studied. The course of PCD events in the different tissues of the developing grain in combination with possible executors of PCD will be delineated.

## Materials and Methods

### Plant material

*Hordeum vulgare* cv. Barke plants were grown in greenhouses (18°C and 16/8 h light/dark regime). Caryopses were harvested in two-day interval and hand-separated into the pericarp and endosperm fractions as described previously [12]. For micro-dissections, whole caryopses were collected and kept at −80°C until use.

### TUNEL assay

TUNEL assay was performed as described [12]. Both negative and positive controls were performed only at 10 DAF. For negative control, TdT was omitted in the reaction. For positive control of the reaction, the sections were treated with DNase (1500 U ml^−1^) prior to labelling with the TUNEL mix (Fig. S1).

### Caspase assay

The samples for caspase assays were homogenized in liquid nitrogen and re-suspended in 2xCASPB buffer (100 mM HEPES, 0.1% CHAPS, 1 M DTT, pH 7.0) at 4°C. Cell debris was separated by centrifugation at 13000 rpm for 10 min at 4°C and the supernatant was used for the reactions or stored at −70°C. Protein concentration in the extracts was estimated by Bradford assay (BioRad, Hercules, CA, USA). Caspase-like activities were measured in 150 µl reaction mixtures containing 25 µg of protein sample and 10 µM of caspase substrate. Caspase-like activities were detected using the following substrates: acetyl-Tyr-Val-Ala-Asp-7-amido-4-methyl coumarin (Ac-YVAD-AMC) for caspase-1 activity; acetyl-Asp-Glu-Val-Asp-7-amido-4-methyl coumarin (Ac-DEVD-AMC) for caspase-3 activity; acetyl-Leu-Glu-Val-Asp-7-amido-4-methyl coumarin (Ac-LEVD-AMD) for caspase-4 activity; acetyl-Val-Glu-Ile-Asp-7-amido-4-methyl coumarin (Ac-VEID-AMC) for caspase-6 activity; acetyl-Ile-Glu-Thr-Asp-7-amido-4-methyl coumarin (Ac-IETD-AMD) for caspase-8 activity; and acetyl-Leu-Glu-His-Asp-7-amido-4-methyl coumarin (Ac-LEHD-AMC) for caspase-9 activity. Emitted fluorescence was measured after one hour incubation at room temperature with a 360 nm excitation wave length filter and 460 nm emission wave length filter in a spectrofluorometer (Spectra Max Gemini, Molecular Devices, U.S.A). Four repetitions were performed for determination of each value and standard deviations were calculated. The system was calibrated with known amounts of AMC hydrolysis product in a standard reaction mixture. Blanks were used to account for the spontaneous breakdown of the substrates. The data were analyzed by one-way analysis of variance (ANOVA) followed by a posthoc-test after Holm-Sidak using Microsoft Excel version 2010, with Daniel’s XL toolbox version 6.10 [57].

To check the specificity of the caspase assays, the specific protease inhibitors were used to suppress the respective caspase-like activity. The following inhibitors were used: Ac-YVAD-CHO to suppress caspase-1 activity, Ac-DEVD-CHO to suppress caspase-3 activity, Ac-LEVD-CHO to suppress caspase-4 activity, Ac-VEID-CHO to suppress caspase-6 activity, Ac-IETD-CHO to suppress caspase-8 activity and Ac-LEHD-CHO to suppress caspase-9 activity. All caspase substrate and inhibitors were purchased from Enzo Life Sciences (Germany). Assays were performed as described above with the addition of the respective inhibitors (20 µM) to the reaction mixture.

### Identification of protease genes with potential caspase-like activity

To identify genes potentially encoding proteases with caspase-like activity, barley full length cDNA data base [32] was screened by BLASTX using already described gene sequences encoding proteases with proven caspase-like activity. The corresponding barley sequences were PCR amplified from a cDNA library of developing grains and re-sequenced using gene-specific primers (Metabion, Germany). Sequence data were processed using the Lasergene software (DNAstar, USA). The phylogenetic trees were built using ClustalW software.

### Tissue preparation for laser micro-dissection and pressure catapulting (LMPC)

Frozen caryopses were transferred to a cryostat kept at −20°C. Using a razor blade, the middle part of the caryopses was cut out and glued onto the sample plate by using O.C.T compound. Sections of 20 µm thickness were cut and immediately mounted on PEN membrane slides (PALM). PEN membrane slides were stored for 7 days in the cryostat at −20°C until complete dryness. Prior to laser-assisted micro-dissection, dry cryo-sections were adapted to room temperature for several minutes. LMPC procedure for isolation of specific grain tissues using the PALM® MicroBeam laser system (PALM) has been performed as described in Thiel et al. [33].

### RNA processing and qRT-PCR

For each sample, RNA was extracted from 30 to 50 sections of isolated tissues using the Absolutely RNA Nanoprep Kit (Stratagene). Total RNA was amplified by one round of T7-based mRNA amplification using the MessageAmp aRNA Kit (Ambion) to generate tissue-specific antisense RNA (aRNA). After quality assessment of aRNA populations first strand cDNA was synthesized using SuperScript III (Invitrogen) with random priming according to the manufactureŕs instructions. The Power SYBR Green PCR master mix was used to perform reactions in an ABI 7900 HT Real-Time PCR system (Applied Biosystems). Data were analyzed using SDS 2.2.1 software (Applied Biosystems). Three replications were conducted for each transcript. The data were analyzed by ANOVA followed by a posthoc-test using Microsoft Excel with Daniel’s XL toolbox version 6.10 [57].

The highest relative expression in the group of genes was taken to 100% and expression of the other genes and stages was re-calculated to that value. Primers used for qRT-PCR are listed in Table S1.

## Results

### Detection of PCD in the developing barley grains by TUNEL assay

**Figure 1:**
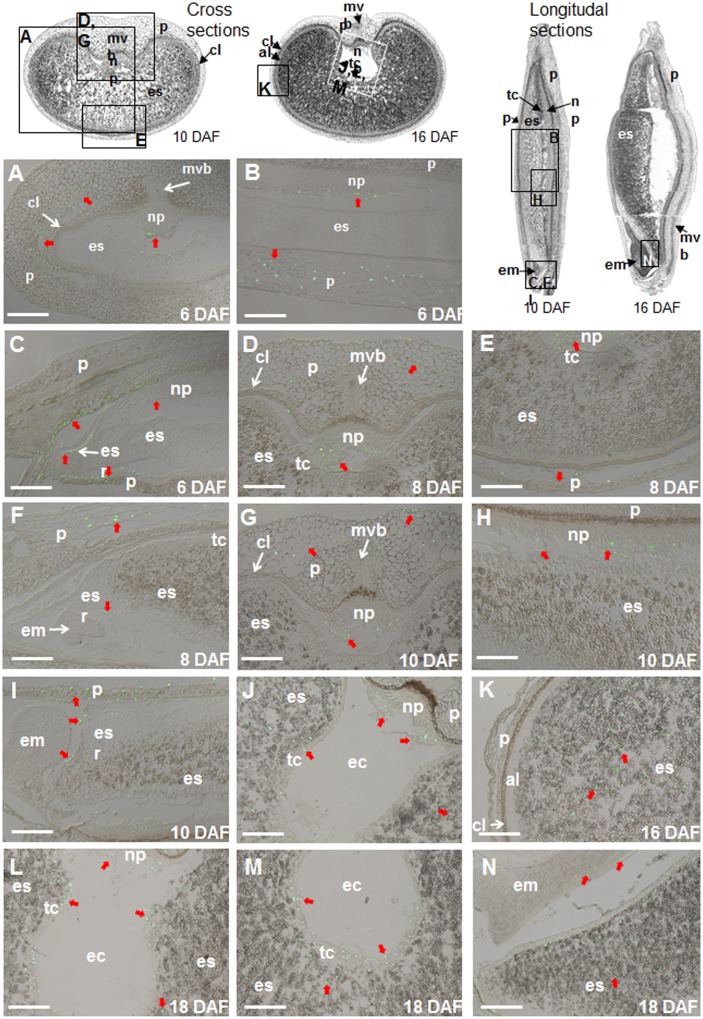
Localization of nuclear DNA fragmentation detected by the TUNEL assay at 6 (A–C), 8 (D–F), 10 (G–I), 16 (J, K), and 18 DAF (L–N). TUNEL-positive nuclei are visualized as green signals and indicated by red arrows. Upper panel demonstrates positions of histological sections used for TUNEL assay at the reconstructed cross and longitudal views of a barley grain. al, aleurone; cl, chlorenchyma, em, embryo; es, endosperm; esr, embryo surrounding region; mvb, main vascular bundle; np, nucellar projection; nu, nucellus; p, pericarp, tc, transfer cells. Bars = 200 µm.

Degradation of DNA and disintegration of nuclei are common features of PCD that can be detected by TUNEL assay. Here, we have analyzed PCD pattern during whole development of the barley grain. Only nuclei of the nucellar cells facing to endosperm were TUNEL-labeled between anthesis and three days after flowering (DAF) (Fig. S1) coinciding with endosperm growth [12]. The nucellus is degenerated around 4 DAF, and further endosperm expansion occurs at the expense of pericarp. Coinciding with this, the first TUNEL-labeling nuclei were visible in the innermost cells of the lateral mesocarp region (Fig. S1). The other tissues, including endosperm and nucellar projection, were free of label. Beginning at 6 DAF, the TUNEL-positive nuclei spread throughout the whole mesocarp layer being especially abundant in lateral and dorsal regions (Fig. 1A, B). The chlorenchyma (endocarp) however did not show any labeled nuclei remaining alive till grain maturation (Fig. 1A, D, G, K). The first labeled nuclei were visible at margins of the nucellar projection (Fig. 1A). Numerous labeled nuclei were also detected in close vicinity to the embryo but not in the embryo itself (Fig. 1C). In this region, large number of TUNEL-positive nuclei at margins of the nucellar projection and the pericarp facing the embryo as well as many nuclei of the embryo-surrounding region (ESR) were labeled (Fig. 1C). ESR is the part of the endosperm, which cellularizes first in development [46]. The other endosperm regions were completely free of label (Figs. 1A–C). With ongoing caryopsis development, the dorsal region of the pericarp becomes largely disintegrated and only few labeled nuclei are visible (Fig. 1E). In contrary, the ventral region of pericarp starts to disintegrate and is filled with numerous TUNEL-positive nuclei (Fig. 1D, G). Disintegrating nuclei were also observed at the margins of the nucellar projection, but not in main vascular bundle, chlorenchyma and starchy endosperm (Fig. 1D–H). However, nuclei of endosperm cells close to the embryo were labeled at 8 DAF as well as 10 DAF (Figs. 1F, I) but not cells of embryo itself. In the late grain filling phase (16 DAF), TUNEL-positive nuclei were still detected in the nucellar projection and the ventral parts of pericarp (Fig. 1J). Numerous labeled nuclei were also visible in different regions of starchy endosperm but not the aleurone layer (Fig. 1K). In addition, some nuclei of the transfer cell layer were TUNEL-positive at 16 DAF (Fig. 1J). Two days later, labeling of nuclei spreads to almost all cells of the transfer cell layer (Fig. 1L, M) besides being also detectable in the starchy endosperm and nucellar projection. At 18 DAF, TUNEL-positive nuclei appeared in the embryo. Especially, almost all nuclei of two cell rows in the scutellum were TUNEL-labeled. Many other TUNEL-positive nuclei were also found in other parts of the embryo (Fig. 1N). Because the embryo cells are small in size, the TUNEL-labeled nuclei in the embryo appear to be smaller compared to other tissues.

No TUNEL labeling was detected in control sections when the TdT enzyme had been omitted. Almost all nuclei were labeled in positive controls, treated with DNase prior to TUNEL assay, demonstrating the validity of the procedure (Fig. S1).

### Caspase-like activities in the pericarp and endosperm fractions of developing barley grains

As plant PCD has been shown to be associated with caspase-like activities, the profiles of YVADase (caspase-1-like), DEVDase (caspase-3-like), LEVDase (caspase-4-like), VEIDase (caspase-6-like), IETDase (caspase-8-like) and LEHDase (caspase-9-like) activities in pericarp and endosperm fractions of the developing caryopses were investigated.

**Figure 2:**
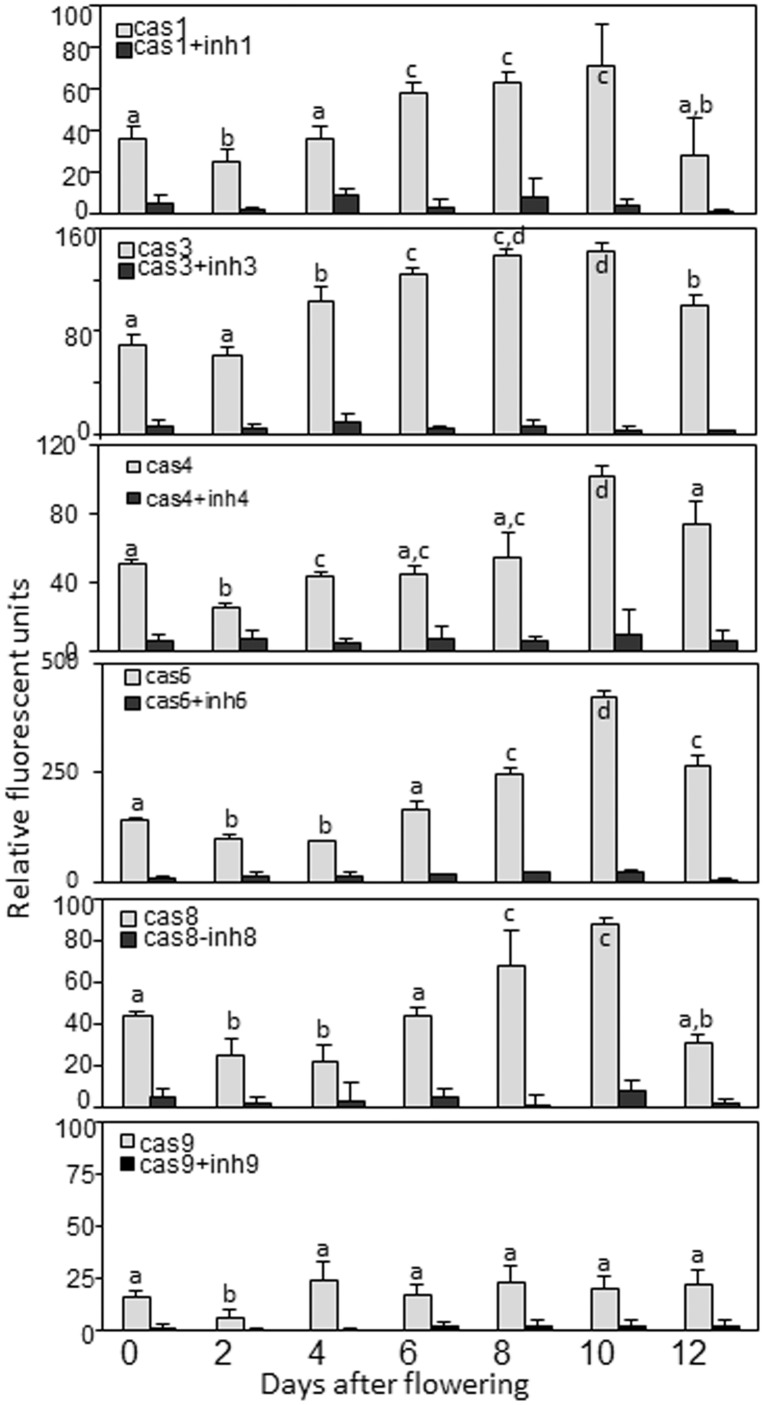
Caspase-like activities and effect of specific caspase inhibitors on corresponding caspase-like activity in barley pericarp. Data are means ± SD, *n* = 4, values followed by the same letter do not differ significantly at *p*>0.05.

In the pericarp, the highest activity was detected with the caspase-6 substrate, Ac-VEID-AMC, followed by caspase-3 (Ac-DEVD-AMC), caspase-4 (Ac-LEVD-AMC), caspase-1 (Ac-YVAD-AMC), and caspase-8 (Ac-IETD-AMC) substrates (Fig. 2). Cleavage activities using all substrates generally increased with aging of pericarp and all peaked at 10 DAF (Fig. 2). The caspase-9-like (Ac-LEHD-AMC substrate) activity was overall low in the pericarp and did not show any developmental pattern. Protease inhibitors, specific for each caspase, strongly inhibited the corresponding caspase-like activity, validating these activities in the pericarp (Fig. 2).

**Figure 3:**
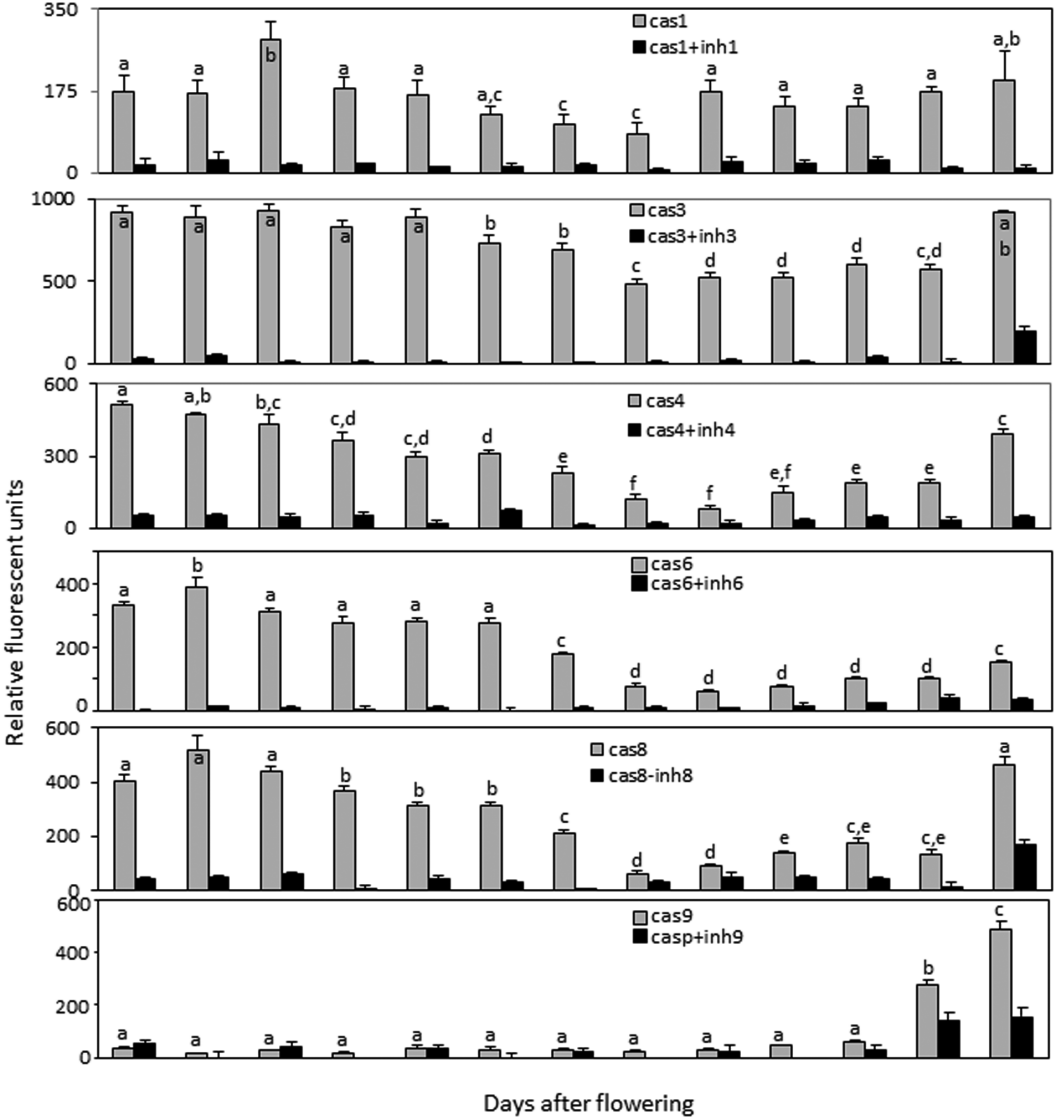
Caspase-like activities and effect of specific caspase inhibitors on corresponding caspase-like activity in the developing endosperm. Data are means ± SD, *n* = 4, values followed by the same letter do not differ significantly at *p*>0.05.

In the developing endosperm fraction, two peaks of caspase-like activities were detected for caspase-1, caspase-3, caspase-4, caspase-6 and caspase-8 substrates. The first prolonged increase in activity was measured between anthesis and 12 DAF which quickly declined thereafter. The second increase in activity was observed during grain maturation starting from 20–22 DAF (Fig. 3). The second increase in caspase-1-like and caspase-6-like activities was not strongly pronounced (Fig. 3). The activities with caspase-9 substrate were barely detectable in the endosperm fraction throughout development except of grain maturation where the strong increase in the activity was detected after 20 DAF (Fig. 3). The specific caspase inhibitors showed inhibitory effects for either caspase-like activity during endosperm development.

To conclude, caspase-1-like, caspase-3-like, caspase-4-like, caspase-6-like and caspase-8-like activities were detected in the developing pericarp, all with increasing profiles towards 10 DAF. Two increases in caspase-like activities were measured in the endosperm fraction with majority of caspase substrates. The increase in caspase-9-like activity was only detected during grain maturation.

### Identification and expression analysis of genes with potential caspase-like activities in barley grains

We described recently seven genes encoding vacuolar processing enzyme (VPE) with potential caspase-1-like activity and found that *HvVPE4* is exclusively expressed in the deteriorating pericarp, *HvVPE2a* (and possibly *HvVPE2b–HvVPE2d*) transcripts are specific for nucellus/nucellar projection and *HvVPE1* is transcribes in late endosperm [12]. Here we analyzed barley genes encoding proteases with potential caspase-3 and caspase-6 activities: β1 and β2 subunits of the 20S proteasome with caspase-3 activity and phytaspase with caspase-6 activity.

**Figure 4:**
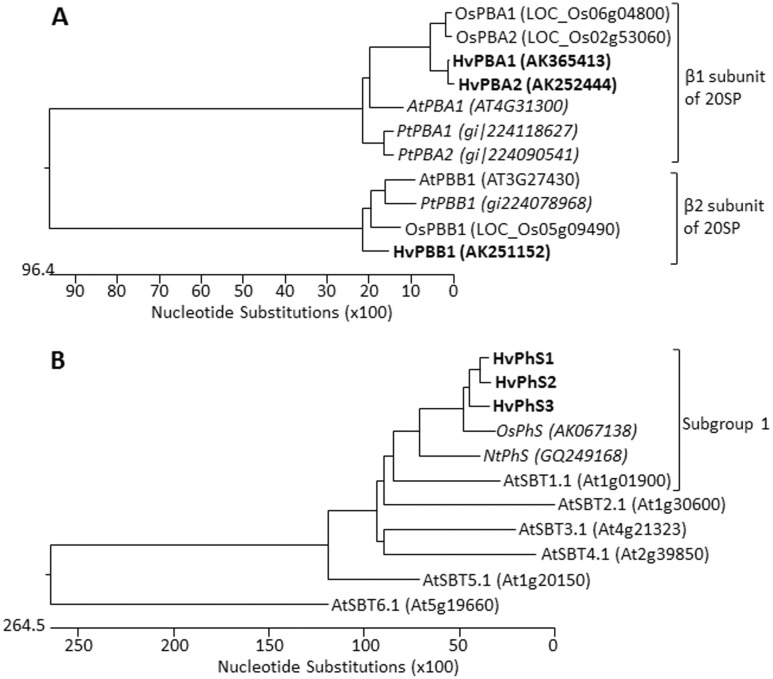
Phylogenetic trees of proteasome subunits PBA and PBB (A) and phytaspases (B) drawn with the ClustalW software. The horizontal scale represents the evolutionary distance expressed as a number of substitutions per amino acid. The putative phytaspases and proteasome subunits PBA1 of barley are shown in bolt. (A) Putative barley (Hv) proteasome subunits PBA and PBB are similar to the corresponding genes from *Arabidopsis thaliana* (At), *Populus tomentosa* (Pt) and *Oryza sativa* (Os). (B) Putative barley (Hv) phytaspases together with phytaspases from *Nicotiana tabacum* (Nt) and *Oryza sativa* (Os) belong to the subgroup 1 of subtilase-like proteases. The phytaspases with proven caspase-6 activity are shown in italic. Only one Arabidopsis subtilase-like protease per subgroup is shown (Rautengarten et al., 2008) in order to simplify the figure.

In Arabidopsis, the 20S proteasome consists of seven α subunits encoded by 12 genes and seven β subunits encoded by 11 genes [35]. However, only β1 (*PBA* gene) and possibly β2 (*PBB* gene) subunits have been shown to exhibit caspase-3 like activity [3], [28]. Therefore, we have searched the barley full length cDNA data base [32] for barley *PBA* and *PBB* genes using homologous poplar sequences [3] as queries. Two genes encoding the putative β1 subunit were found in the barley cDNA data base, the same number as found in poplar and rice while only one gene encodes PBA in Arabidopsis (Fig. 4A). HvPBA1 and HvPBA2 are almost identical at amino acid level (95.5% identity) and very similar to other known PBAs sharing 75.5–76.4% identity to AtPBA, 74.8–75.6% to both poplar PBA proteins and 85.7–89.5% identity to putative OsPBA1 and OsPBA2 sequences indicating that the genes encoding PBA are very conserved in plants.

Only one gene encoding β2 subunit was found in barley as well as in Arabidopsis, poplar and rice (Fig. 4A). The deduced HvPBB amino acid sequence is very similar to the other plant counterparts with an identity ranging from 75.7% (Arabidopsis) to 87.9% (rice).

Some members of huge family of subtilisin-like proteases have been shown to possess caspase-6-like activity [29] and are called phytaspases (PhS). Using rice phytaspase [29] as a reference, three putative barley phytaspase genes have been selected from the full length cDNA database [32]. Barley phytaspases share 77.5–90.2% identity to each other and 68.0–73.7% identity to OsPhS at the amino acid level. The deduced HvPhS1–HvPhS3 proteins group together with tobacco and rice phytaspases and all belong to the subgroup 1 of the subtilisin-like proteases (Fig. 4B).

**Figure 5:**
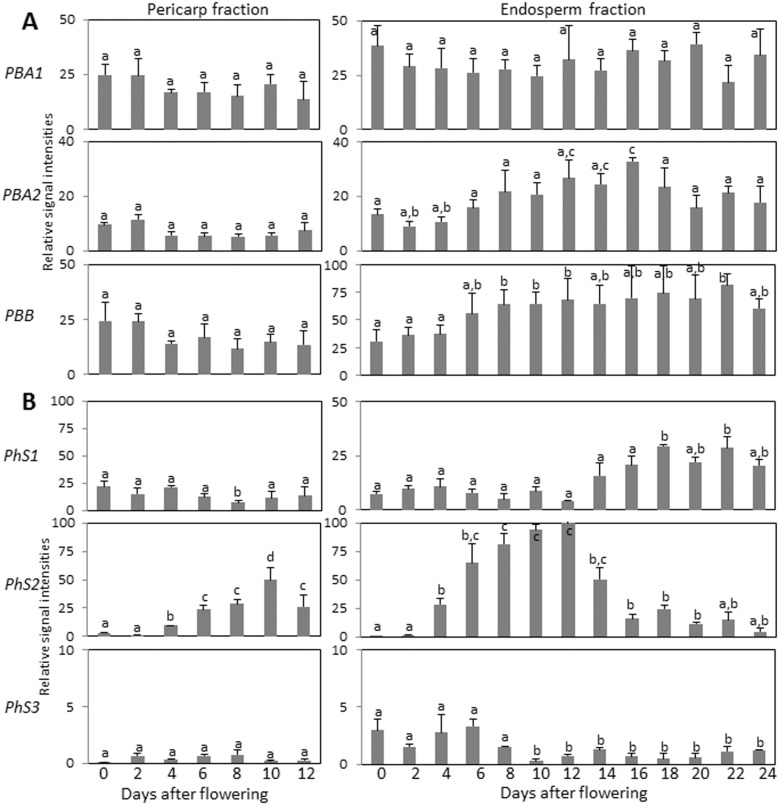
Transcript profiling of the proteasome subunits *PBA* and *PBB* (A) and phytaspase (*PhS*) genes (B) in pericarp (left) and endosperm fractions (right) of the developing barley grains determined by real-time quantitative RT-PCR analysis. Data are means ± SD, *n* = 3, values followed by the same letter do not differ significantly at *p*>0.05.

Gene expression patterns were determined in manually isolated pericarp and endosperm fractions of barley grains between anthesis and 24 DAF by quantitative reverse transcription PCR (qRT-PCR). Both *HvPBA1* and *HvPBA2* as well as *HvPBB* genes did not show developmental expression profile in the pericarp (Fig. 5A). In endosperm fraction, *HvPBA1* ubiquitously expressed while *HvPBA2* and *HvPB* display weak increase in transcription during grain filling (Fig. 5A). The *HvPHS1* gene was also ubiquitously expressed in the pericarp (Fig. 5B) while *HvPhS3* transcripts were barely detected in the tissue. Solely the *HvPhS2* transcripts accumulated in the pericarp with increasing abundance towards 10 DAF and declining afterwards (Fig. 5B). In the endosperm fraction, the *HvPhS1* transcripts were detected at low levels during early grain development but exhibited increase in expression during later grain filling phase starting from 16 DAF (Fig. 5B). The relative expression of *HvPhS2* was the highest in the endosperm fraction among the three barley phytaspases and peaks between 4 and 12 DAF decreasing thereafter (Fig. 5B). Contents of *HvPhS3* mRNA were low in the endosperm fraction throughout development however with weak increase at early developmental stages (Fig. 5B).

**Figure 6:**
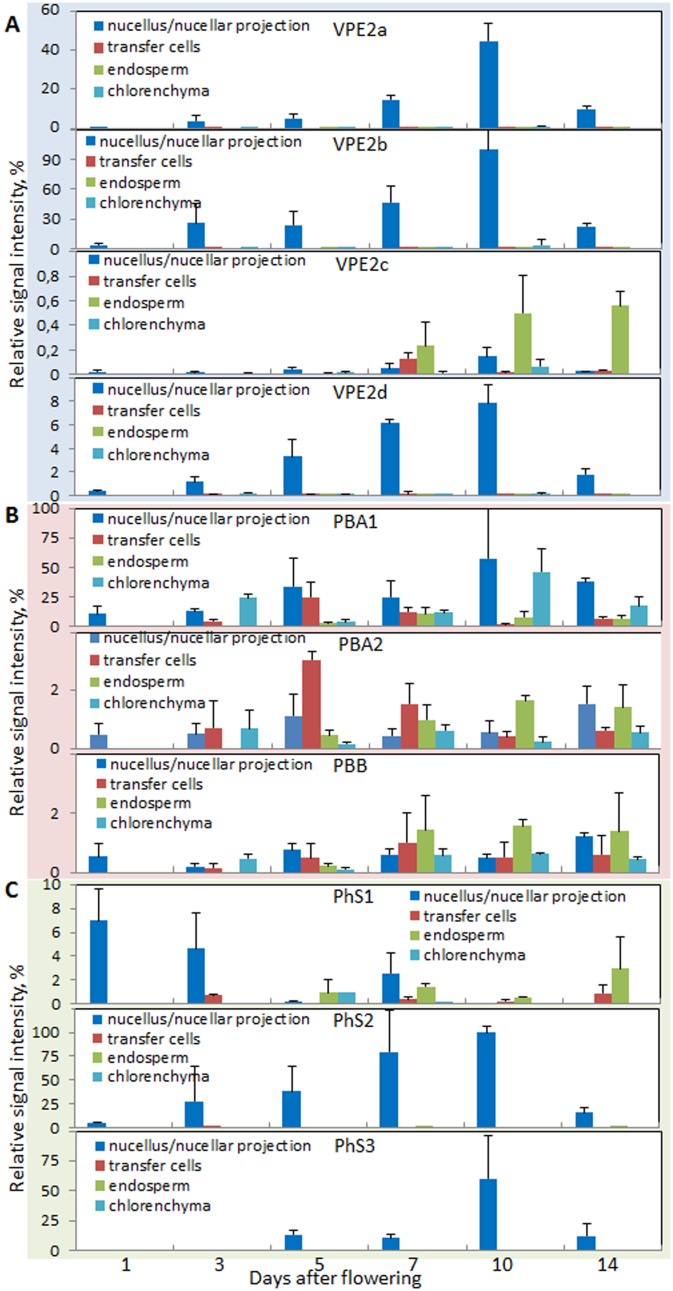
Expression profiles of the vacuolar processing enzymes VPE2a-VPE2d (A), proteasome subunits PBA and PBB (B) and phytaspase (PhS) genes (C) in the different tissues micro-dissected from the developing barley grains.

The pericarp fraction used for qRT-PCR encloses only maternal tissues consisting predominantly of mesocarp and epidermis (exocarp). Endosperm fraction, however, represents a complex sample consisting of filial and maternal tissues in changing proportions at different developmental stages and encompasses the filial endosperm itself, endosperm transfer cells, aleurone and embryo surrounding region but also maternal nucellus/nucellar projection and chlorenchyma (endocarp). Therefore, to study the tissue-specific gene expression profiles we used micro-dissected samples of these tissues from grains at different developmental stages. Because the gene expression of the vacuole processing enzymes *HvVPE2b–HvVPE2d* in nucellus/nucellar projection was not experimentally proven [12], we analyzed also their transcript abundances in micro-dissected tissues. As expected, the expression of *HvVPE2a*, *HvVPE2b* and *HvVPE2d* was found exclusively in nucellus and nucellar projection with a maximum between 7 and 10 DAF (Fig. 6A). *HvVPE2b* gene activity was the highest among all VPEs expressed in these tissues. Accumulation of *HvVPE2a* transcripts was two-fold lower as of *HvVPE2b*, and transcript levels of *HvVPE2d* reached only one tenth of those for *HvVPE2b*. Expression of *HvVPE2c* was detected only at basic level (less than 1% of *HvVPE2b*) in all studied tissues (Fig. 6A) confirming previous data [12]. Expression of *HvPBA1*, *HvPBA2* and *HvPBB* genes was detected in all micro-dissected tissues analyzed showing neither preference for any tissue nor characteristic developmental profile (Fig. 6B). Among the three phytaspase genes, *HvPhS2* expression was the highest and found exclusively in the nucellar projection depicting a maximum of expression at 10 DAF (Fig. 6C). *HvPhS3* transcripts were also detected specifically in the nucellar projection peaking around 10 DAF albeit at lower expression level (Fig. 6C). Expression of *HvPhS1* was observed at relatively low levels in all analyzed micro-dissected tissues without clear developmental profile (Fig. 6C).

To conclude, the *HvPBA1*, *HvPBA2*, *HvPBB*, *HvPhS1* and *HvPhS3* are expressed without certain developmental patterns in pericarp. The expression of *HvPhS2* is increased at later stages of pericarp development. Abundance of *HvPBA2* and *HvPBB* mRNAs are weakly increased during grain filling. The transcripts of *HvVPE2a*, *HvVPE2b*, *HvVPE2d*, *HvPhS2* and *HvPhS3* are detected exclusively in the nucellar tissues of the developing barley grains. The *HvPhS1* mRNA abundances increase in the maturating endosperm.

## Discussion

Programmed cell death (PCD) is an essential part of the life of any multicellular organism. PCD plays a crucial role in tissue and organ development and in the maintenance of the cellular homeostasis of a tissue. In this work we analyzed PCD events in the developing barley caryopsis. Activation of caspases is a hallmark of apoptosis and inflammatory response in animals [17], [36]. Caspase-like activities become also markedly enhanced upon induction of PCD in plants [19], [23]. In both pericarp and endosperm fractions of developing grains, distinct caspase-like activities showed similar profiles albeit their relative activity levels were different. Activities with all tested caspase substrates excluding LEHD (caspase-9 substrate) increase during pericarp development (Fig. 2) coinciding with the abundance of TUNEL-positive nuclei (Fig. 1) and degradation of the pericarp tissue [12]. Increased activities with almost all caspase substrates except of caspase-9 were detected in the endosperm fraction during early development (Fig. 3). The second increase in all caspase-like activities including caspase-9-like was found during grain maturation (Fig. 3). Based on these observations we tend to conclude that coaction of caspase-like protease activities may execute and regulate PCD processes in plant tissues similar to that occurring in animal cells [17], [37]. In animals, the caspases are classified into inflammatory, apoptotic initiator and apoptotic effector groups [36]. The latter group is processed and activated by upstream caspases and performs downstream steps cleaving multiple cellular substrates. The effector caspases are usually more abundant and active than initiator caspases [36]. In the barley grains, caspase-6-like activity is highest in both pericarp and early endosperm fractions followed by the caspase-3-like activity (both effector activities in animals) while caspase-8-like and especially caspase-9-like activities were substantially lower (Figs. 2, 3). Referring to the animal model, it is tempting to speculate that proteases with caspase-6-like and caspase-3-like activities may fulfill effector role in plant PCD while proteases with caspase-8-like and caspase-9-like activities are PCD initiators. Caspase-like proteases executing PCD may differ among distinct plant tissues. For instance, the caspase cascade in the pericarp and early endosperm fractions may not include caspase-9-like activity, because the latter has been barely measurable in these tissues. In contrast, the potential caspase coaction in the maturing endosperm includes caspase-9-like activity but caspase-6-like activity may play minor role (Fig. 3). The possible coaction of proteases with caspase-like activities in acquisition and execution of plant PCD needs further experimental confirmation.

We detected the caspase-4-like activity in plants for the first time. Its patterns of activity in both pericarp and endosperm (Figs. 2, 3) coincide with the degeneration processes in the respective tissue (Fig. 1). The specific protease inhibitor could strongly inhibit the caspase-4-like activity. The specific protease responsible for the newly detected caspase-4-like activity remains to be detected.

**Figure 7:**
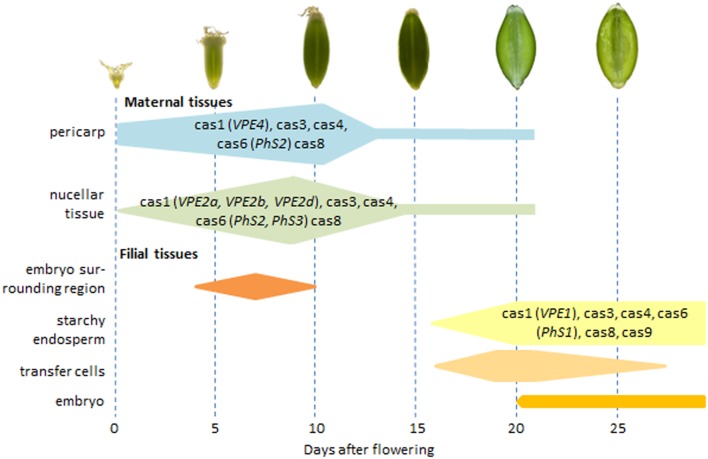
Scheme illustrating programmed cell death processes together with potentially involved activities and genes (in brackets) in distinct tissues of the developing barley grains. Activities: cas1, caspase-1-like; cas3, caspase-3-like; cas4, caspase-4-like; cas6, caspase-6-like; cas8, caspase-8-like; cas9, caspase-9-like. Genes: VPE, vacuolar processing enzyme; PhS, phytaspase.

The expression of the *HvVPE* and *HvPhS* genes largely coincides with PCD of the respective tissue (Fig. 7) (see also below). However, none of the genes encoding β1 or β2 subunits of the 20S proteasome shows a specific developmental profile (Figs. 5, 6). However, caspase-3-like activity, potentially mediated by the corresponding proteins [3], [28], displays developmental pattern of the activity in barley grains (Figs. 2, 3). It might be possible that plant β1 or β2 subunits of 20S proteasome subunit are post-translationally regulated to control the PCD. The 20S proteasome subunit as part of the ubiquitin/26S proteasome complex plays a role in nearly all processes of plant development by selectively eliminating regulatory proteins [41] and, therefore, its activity has to be fine controlled. It is also possible that other proteases display caspase-3-like activity in barley grains.

PCD processes in the distinct grain tissues are summarized in Fig. 7 and discussed below in more details.

### Programmed cell death in the nucellus and nucellar projection

Nucellus is the first tissue undergoing PCD after beginning of caryopsis development (besides the antipodals and synergid cells, which however belong to gametophyte). The first TUNEL-labeled nuclei are visible at the margins of nucellus facing developing endosperm very soon after fertilization in both barley (Fig. S1) and wheat [10]. With the endosperm growth, PCD in nucellus expands to outward cell layers finally resulting in complete disappearing of the nucellus till 4–5 DAF except the cells adjacent to the main vascular bundle, which develop to nucellar projection [12], [14]. The nucellar projection together with the opposite endosperm transfer cells operate as a main conduit for nutrient supply from the main vascular bundle to endosperm [11], [13]. The first TUNEL-positive nuclei appear at margins of the nucellar projection facing the endosperm transfer cells around 6 DAF (Fig. 1). Thereafter the degenerating nuclei at margins of the nucellar projection are detectable till late grain maturation (Fig. 1). Permanent cell turnover seems to occur in the nucellar projection. New cells are produced in the mitotic region, then cells elongate, produce thick cell walls [41] and become functionally active before they degenerate and thereby direct cell content and cell remnants into the apoplastic space. This mechanism of nutrient delivery is not fully understood, despite its importance for endosperm filling and grain yield [42]. There are no symplastic connections between nucellar projection and endosperm transfer cells, and nutrient transport across maternal/filial border occurs apoplastically [41]. As deduced from thick cell walls of elongating cells [41] and expression of many transporters [14], the nutrient transfer through nucellar projection involves both symplastic and apoplastic pathways and evidently requires PCD at the site of nucellar projection [11], [14]. Disruption of PCD in nucellar tissues affects endosperm development and grain weight in barley [11] and rice [43].

Because hand-isolated endosperm fraction always includes nucellar projection [12], [34], the first increase in almost all caspase activities in the endosperm fraction (Fig. 3) may be related at least in part to PCD of nucellus and nucellar projection. The increase in caspase-1-like activity may be acquired by HvVPE2a, HvVPE2b and HvVPE2d proteases which exclusively expressed in nucellus and nucellar projection (Fig. 6). The caspase-1-like activity for HvVPE2b (HvLeg2) has been already proven [27]. The expression patterns of the *HvPhS2* and *HvPhS3*, which are exclusively active in the nucellar projection (Fig. 6), coincide to the caspase-6-like activity profile in the early endosperm fraction (Fig. 3) indicating that HvPhS1 and HvPhS2 may be responsible for the caspase-6-like activity.

### Programmed cell death in pericarp

After nucellus degeneration, the endosperm enlarges by cost of pericarp cells which undergo PCD starting from the innermost cell layer of mesocarp between 4 and 5 DAF, as seen from distribution of TUNEL-positive nuclei [12]. The lateral and dorsal parts of the mesocarp disintegrate already till 10–12 DAF (Fig. 1E) whereas the ventral region around the main vascular bundle persists undergoing later a gradual degeneration until grain maturation (Fig. 1J). The green and photosynthetically active chlorenchyma layer [44] however does not show any TUNEL-positive signals during observation time (Fig. 1). Probably this layer disintegrates during desiccation when the maturating grain turns from green to yellow. Obviously, the chlorenchyma plays important role for caryopsis development. Perception of light by photosynthetically active seed layer is thought to represent a strategy to sense environment and provide a means of tuning grain metabolism according to the changing conditions [45].

Coinciding with PCD progression in the pericarp, we detected increase of the caspase-1-like, caspase-3-like, caspase-4-like, caspase-6-like and caspase-8-like but not caspase-9-like activities towards 10 DAF and their decline thereafter (Fig. 2). The transcript profile of early described mesocarp-expressed *HvVPE4* gene [12] coincides with pattern of caspase-1-like activity (Fig. 2) further supporting that HvVPE4 may be responsible for the activity. The profile of caspase-6-like activity (Fig. 2), expression of *HvPhS2* gene (Fig. 5) and the patterns of TUNEL-positive nuclei (Fig. 1) also coincide indicating that HvPhS2 may be involved in PCD as protease with caspase-6-like activity in the pericarp.

### Programmed cell death in the endosperm

Early endosperm develops by divisions of nuclei without cytokinesis resulting in the endosperm coenocyte [46]. Coencyte begins to cellularize around 4 DAF in the embryo surrounding region (ESR) [46], [47]. Transfer cell layer is also formed at this time [33]. TUNEL-labeling nuclei in the endosperm are absent between anthesis and 6 DAF indicating that cell degradation processes do not occur during early endosperm development. No genes potentially encoding proteases with caspase-1-like and caspase-6-like activities are expressed in the early developing endosperm (Fig. 6). Transfer cells are also free of the corresponding transcripts (Fig. 6). Therefore, the increase in almost all caspase-like activities in early developing endosperm fraction (Fig. 3) is likely not due to PCD processes in endosperm but may be related to PCD in the nucellar tissues as described above. The first degenerating nuclei appeared in cellularized ESR already 6 DAF (Fig. 1C). Therefore, the high value of caspase-like activities between 4 and 12 DAF in the endosperm fraction may be at least in part correspond to PCD in ESR as well.

PCD of ESR in maize and wheat has been described at histological level many years ago [48], [49]. Here, we document nuclei degradation in barley ESR shortly after cellularization starting from the cells facing the embryo (Fig. 1C, F). The ESR can be subdivided into three different regions distinguished by vacuole size and degree of cellular vacuolization [47]. The highly vacuolated cells facing the embryo degrade firstly followed by the deeper cell layers. This pattern is reminiscent to that of the nucellar projection [14] where the degrading cells at the margins contribute to nutrient transfer to the endosperm [11]. In analogy, we hypothesize here that PCD of ESR cells is important for the nutrient supply to the embryo releasing cell contents and cell remnants into liquid-filled embryonic space. Besides, PCD of ESR provides space for the growing embryo. In the embryoless mutants of maize, the endosperm develops a normal-sized embryo cavity suggesting the existence of an intrinsic program for ESR formation independent from embryo development [51]. PCD of the ESR may be a part of such a program. The nuclei of pericarp cells surrounding the embryo from maternal side are also strongly labeled in TUNEL assay. The degradation of the pericarp mainly occurs in the layer adjacent to the embryo and endosperm (Fig. 1C, F, I). We suppose that growing embryo requires space not only of degenerating ESR but also from the maternal pericarp. It is rather unclear whether degrading pericarp cells also contribute to nutrient delivery to the embryo. The direct nutrient supply from pericarp to the embryo can be anticipated, because nucellar projection and transfer cells are still not developed in the embryo region at this developmental stage (Fig. 1F) [50].

With the establishment of transfer cells and endosperm cellularization, the endosperm serves for accumulation of storage compounds. Highly energetic biosynthesis of starch and storage proteins requires intact and metabolically active cells which have be able to convert large amounts of metabolites into storage compounds. This might be reflected in general decrease of caspase-like activities during main filling phase (10–18 DAF) and absence/low expression of related proteases (Figs. 3, 5). With the decline of storage synthesis, the endosperm cells of maize, wheat and rice undergo PCD [15], [16], [52]. The numerous TUNEL-positive nuclei in starchy endosperm of barley grains are visible starting from 16 DAF (Fig. 1J–N). The expression of *HvVPE1* [12] and *HvPhS1* (Fig. 5) are increased during seed maturation coinciding with the increase of caspase-1-like and caspase-6-like activities (Fig. 3). It is tempting to speculate these phytaspase and vacuolar processing enzyme are responsible for corresponding activities in maturating endosperm and required for its PCD. A second increase of caspase-4-like and caspase-8-like activities and unique increase in caspase-9-like activity have been also detected during grain maturation (Fig. 3) albeit the corresponding proteases are still unknown.

Some nuclei of the transfer cell layer are also labeled in TUNEL assay at 16 DAF (Fig. 1K). At 18 DAF, almost all nuclei of the transfer cells are positive in the TUNEL assay, indicating massive cellular disintegration. The transfer cells disintegrate after completion of storage product accumulation and thereby interrupt the delivery of nutrients to the starchy endosperm. Such breakdown of metabolite flow may serve as a signal to endosperm cells for switching from storage product accumulation to maturation and grain desiccation.

### Detection of PCD in the developing embryo

The zygote developing to the embryo starts to divide later than the fertilized central cell which gives raise to the endosperm. Following cell divisions in the embryo are slower than as in syncytial endosperm. No nuclei degradation in the embryo was detected during early development (6–10 DAF; Fig. 1C, F, I). However, almost all nuclei in two cell layers of the scutellum and occasional nuclei in other parts of the embryo were TUNEL-positive at 18 DAF (Fig. 1N), indicating massive tissue reorganization during embryo maturation. It is well known that the scutellum is the last grain tissues undergoing PCD in course of germination after accomplishing the supply of nutrients from the starchy endosperm to the growing embryo [53]. Cell disintegration during embryo development in dicots plants is also a well described phenomenon. After the first division of the zygote, the apical daughter cell gives rise to the embryo proper, while the basal cell develops into the suspensor. The latter is a terminally differentiated structure that is removed by PCD [54]. We have detected for the first time the cell disintegrative processes in the late developing embryo and scutellum of grasses. It is possible that such cell disintegration is a result of scutellum reorganization from supporting tissue for developing embryo to feeding tissues for growing embryo during germinating. The molecular mechanisms responsible for PCD in the late embryo as well as its role in embryo development remain to be studied.

To conclude, the spatial and temporal distribution of the TUNEL-positive nuclei suggests that each seed tissue follows individual pattern of development and disintegration, which however harmonizes with growth of the other tissues in order to achieve proper caryopsis development. In analogy to animal system, programmed cell death in the developing barley caryopsis may require a coaction of caspase-like activities. Expression of distinct genes encoding vacuolar processing enzyme and phytaspase largely coincides with caspase-1-like and caspase-6-like activities in the respective tissue and may be responsible for either caspase activity. However, all above assumptions require experimental confirmations. Due to striking similarity of grain development in barley and wheat as well as in other small grain crops, the results and conclusions about PCD in the barley grains may have impact on research of other important cereal crops.

## Supporting Information

Figure S1 **Negative (A–C) and positive controls (D–F) of TUNEL assay, and standard TUNEL assay performed at 10 DAF (G–H) as well as the localization of nuclear DNA fragmentation detected by the TUNEL assay at 1 (J), 3 (K), 5 DAF (L).**
(TIF)

Table S1 **Primers used in real-time RT-PCR analyses.**
(DOCX)
