# Supplementary figures and images for: Caspase-Like Activities Accompany Programmed Cell Death Events in Developing Barley Grains
Source: PLoS One. 2014 Oct 6;9(10):e109426. doi: 10.1371/journal.pone.0109426 (PMC4186829; doi:10.1371/journal.pone.0109426)

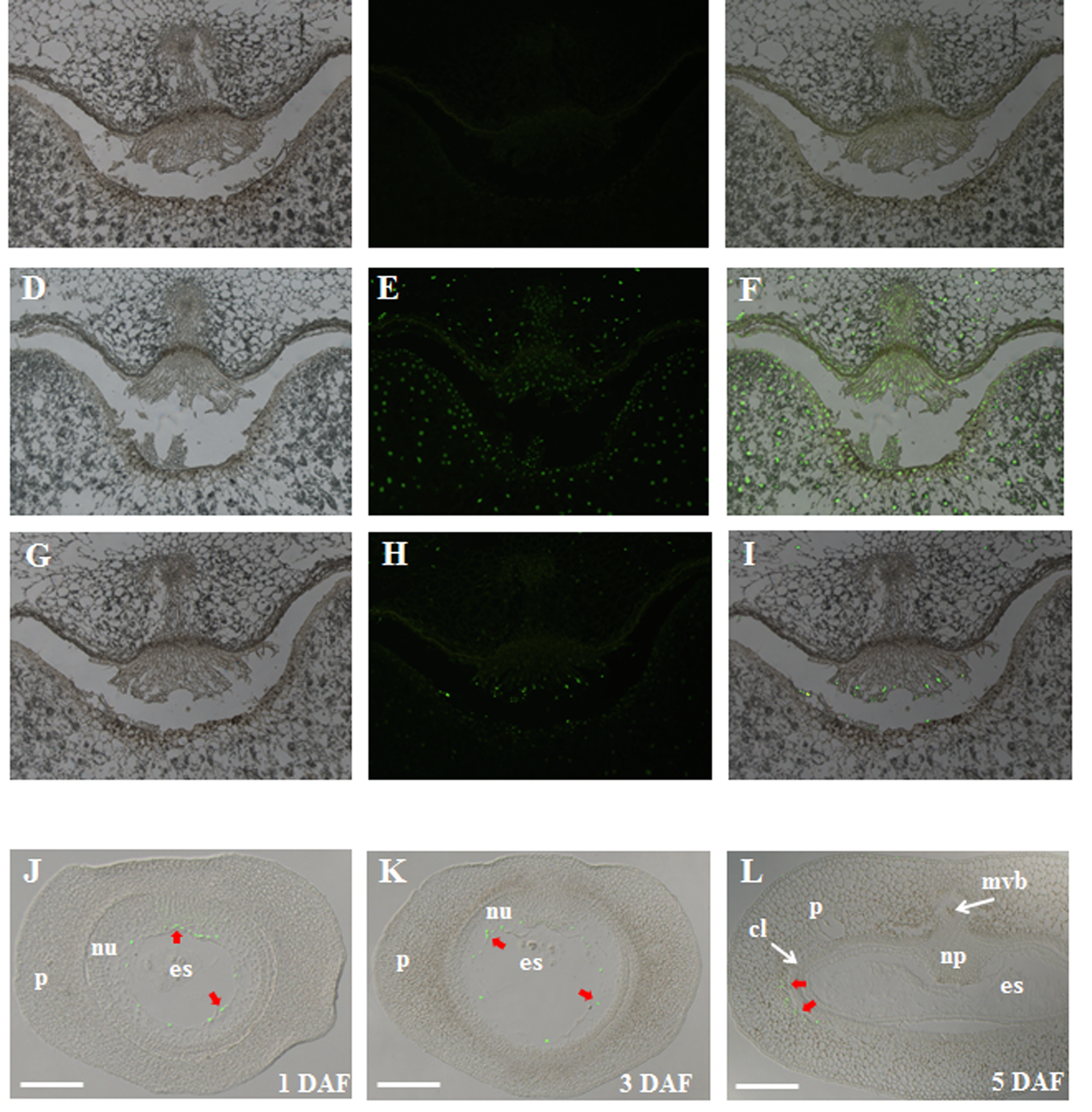

Supplement: Figure S1 — Negative (A–C) and positive controls (D–F) of TUNEL assay, and standard TUNEL assay performed at 10 DAF (G–H) as well as the localization of nuclear DNA fragmentation detected by the TUNEL assay at 1 (J), 3 (K), 5 DAF (L). (TIF) [file pone.0109426.s001.tif]
