# Supplementary material for: Caspase-Like Activities Accompany Programmed Cell Death Events in Developing Barley Grains
Source: PLoS One. 2014 Oct 6;9(10):e109426. doi: 10.1371/journal.pone.0109426 (PMC4186829; doi:10.1371/journal.pone.0109426)
Supplement: Table S1 — Primers used in real-time RT-PCR analyses. (DOCX) [file pone.0109426.s002.docx]

**Supplemental Table S1.**  Primers used in real-time RT-PCR analyses

| **Gene** | **Primer name** | **Sequence** | **Product size** |
| --- | --- | --- | --- |
| *VPE2a*  *VPE2b*  *VPE2c*  *VPE2d*  *PBA1*  *PBA2*  *PBB*  *PhS1*  *PhS2*  *PhS3*  *Actin** | VPE2a_u VPE2a_r  VPE2b_u  VPE2b_r  VPE2c_u  VPE2c_r  VPE2d_u  VPE2d_r  PBA1_f  PBA1­_r  PBA2_f  PBA2_r  PBB1_f  PBB1_r  PhS1_d1  PhS1_r1  PhS2_d2  PhS2_r2  PhS3_d1  PhS3_r1  actin_u  actin_r | 5´-TGCGCTGCAGTACACGGAA-3´  5´-TCTAGCTAGCTAGGAACCTCCG-3´  5´-CAGCGCTTGCAACGGCTACGA-3´  5´-TGCAAGCGGATCAGGGCTGTG-3´  5´-GCGTCTCTGAGGCCCAAATGA-3´  5´-TTATAACCGCCGCAAGCACTGAT-3´  5´-GCTGCCTTTGCCCATCCTG-3´  5´-TCCCCCGTTTAACTGCTCATACTT-3´  5’-AAATGAGGAGGGCGTTAAGAGGAG-3’  5’-GGGAAGCACGGCAAATATCACTGG-3’  5’-GCGGCGGAACCCCCACAC-3’  5’-GAGCGAGGCGGCGTCCAT-3’  5’-ATGGCCGGATCGATGGAACTC-3’  5’-CCTCGGTCGCCCTTGTGTCTG-3’  5’-GCGCATCAATTCGTTGGAGGAA-3’  5’-TTGGCGTATGGTCAGTTGTTGG-3’  5’-CAAAGGCATCCATCAAGTTCGGG-3’  5’-TTGGCAAACAGCGACTGGGG-3’  5’-GCATCGTGCGTGCAAACATC-3’  5’-TGCGCACGTATTCGCGTATGCT-3’  5’-CTGGTTTCGCTGGAGATGATGC-3’  5’-CCGAGGGCGACCAACTATGC-3’ | 57  72  51  91  147  96  181  86  202  57  62 |

** *Hordeum vulgare* *actin* gene (Acc. Nr. AY145451).
